# Sex Differences in Cancer Immunotherapy Efficacy, Biomarkers, and Therapeutic Strategy

**DOI:** 10.3390/molecules24183214

**Published:** 2019-09-04

**Authors:** Shixiang Wang, Li An Cowley, Xue-Song Liu

**Affiliations:** 1 School of Life Science and Technology, ShanghaiTech University, Shanghai 201203, China; 2 Shanghai Institute of Biochemistry and Cell Biology, Chinese Academy of Sciences, Shanghai 200031, China; 3 University of Chinese Academy of Sciences, Beijing 100864, China; 4 St Hugh’s College, The University of Oxford, Oxford 01865, UK

**Keywords:** cancer immunotherapy, immune checkpoint inhibitor, sex differences, tumor mutational burden, biomarker

## Abstract

Sex differences in innate and adaptive immune responses are known, and women generally mount a stronger immune response than men. Cancer immunotherapy, represented by immune checkpoint inhibitors (ICIs), has revolutionized the treatment of cancer, and sex differences in cancer immunotherapy are just starting to be revealed. Here, we summarize recent research progress concerning sex differences in cancer immunotherapy efficacy. On their own, ICIs tend to be more effective in male cancer patients compared with female patients, while ICIs combined with chemotherapy tend to be more effective in female patients than male patients. Male tumors are usually more antigenic than female tumors, and this is reflected by their increased number of tumor mutations and cancer germline antigens. The biomarker tumor mutational burden (TMB), which reflects tumor antigenicity, is more effective at predicting immunotherapy response for female lung cancer patients than for male patients. In this review, we propose different therapeutic strategies for the different sexes: For male cancer patients, the immune environment should be enhanced, whereas for female cancer patients, tumor antigenicity should be enhanced.

## 1. Introduction

Sex is defined by the differential organization of chromosomes, reproductive organs, and sex steroid levels in men and women. This differs from gender, which depends upon behaviors and activities that are determined by society or culture in humans. Sex is a known variable that affects both innate and adaptive immune responses [1]; however, fewer than 10% of immunology-related publications analyze their data considering the sex of the animal or human subjects [2]. In immunotherapy clinical trials, women are still underrepresented compared with men [2]. This is probably because men are frequently used to represent the human species due to historical reasons and there is a concern that the cyclic hormonal changes in a woman’s body may influence the results of clinical trials. However, it would be wrong to assume that the results from male patients apply to female patients and vice versa; therefore, cancer immunotherapy preclinical and clinical studies should be focused on detecting sex differences, in accordance with the sex and gender equity in research (SAGER) guidelines [3].

This review outlines the recent progress on sex differences in cancer immunotherapy, with a specific focus on efficacy of immune checkpoint inhibitor (ICI) therapy, performance of predictive biomarkers, and therapeutic strategies. To compile this review, we searched PubMed and Google Scholar for studies (until Aug 20, 2019) concerning sex differences in cancer immunotherapy. The following keywords were used in the literature searches: “sex differences” or “gender differences” or “hormone” or “estrogen” or “androgen” AND “immunotherapy” or “immune checkpoint inhibitor” or “nivolumab” or “BMS-936558” or “pembrolizumab” or “MK-3475” or “atezolizumab” or “MPDL3280A” or “durvalumab” or “MEDI4736” or “avelumab” or “MSB0010718C” or “BMS-936559” or “cemiplimab” or “REGN2810”. For each article that was identified, the included citations and references were also reviewed.

## 2. Sex Differences in Immune Response

**Figure 1:**
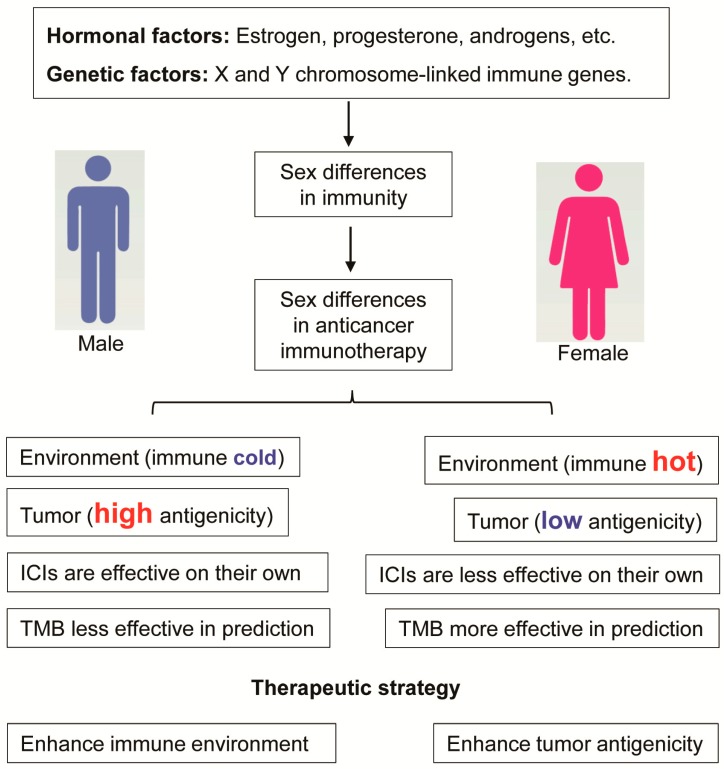
Sex differences in cancer immunotherapy. Women generally mount a stronger immune response than men. These sex differences in the immune response are a result of hormonal differences and differences in sex chromosome genes and can have an impact on cancer immunotherapy efficacy, biomarkers, and, potentially, therapeutic strategy. Male tumors are usually more antigenic than female tumors, and immune checkpoint inhibitors (ICIs) on their own are more effective in males than in females. The tumor mutational burden (TMB) is less effective in response prediction in males than in females. Different therapeutic strategies are proposed for different sexes: For male cancer patients, the immune environment should be enhanced, whereas for female cancer patients, tumor antigenicity should be enhanced.

Generally, women mount a stronger immune response compared to men, and this is reflected in the following aspects of their health (Figure 1).

Autoimmune diseases: In the United States, women are 4 times more likely to develop an autoimmune disease compared with men [4]. This increased incidence in females is especially pronounced in some types of autoimmune disease, such as Sjögren syndrome, systemic lupus erythematosus, thyroid diseases, scleroderma, and myasthenia gravis [5]. Similarly, in both the experimental autoimmune encephalomyelitis mouse model of multiple sclerosis and the non-obese diabetic (NOD) mouse model of spontaneous type I diabetes, female mice show higher incidences and severity of disease compared with male mice [6].

Cancer: For the majority of cancer types, men show a higher risk of malignancy throughout their lifetimes compared with women [7]. Additionally, males have an almost twofold greater risk of mortality from all malignant cancers than females do, with sex-differential outcomes being greatest for larynx, esophagus, bladder, and lung cancers [8]. This male-biased mortality is hypothesized to reflect differences in cancer etiology; however, sex differences in immune function have also been hypothesized to be one of the reasons for this.

Infectious diseases: Enhanced immune response to pathogens among women contributes to an overall lower intensity and prevalence of many infections in women compared with men. However, the increased immune response also increases infectious disease symptoms and severity among women compared with men [9].

Vaccines: Antibody responses to bacterial and viral vaccines are often higher in women than men. This means that the effective vaccine dose is lower for women.

As described, females show a stronger immune response than males. The following mechanisms may contribute to this difference (Figure 1).

Sex hormones: Immune system regulation in health and disease is influenced by sex hormones, including estrogen, progesterone, and androgens, whose levels vary between men and women. For example, the levels of estradiol (E2), a type of estrogen, are variable during the menstrual cycle, high during pregnancy, and low after menopause in females. Estrogen receptors are expressed in various lymphoid tissue cells, including lymphocytes, macrophages, and dendritic cells. E2 enhances both cell-mediated and humoral immune responses [10,11,12,13]. Progesterone, another sex hormone, is produced by the corpus luteum during the menstrual cycle and in high quantities by the placenta during pregnancy. Progesterone receptors are present on many different types of immune cells, including NK cells, macrophages, dendritic cells, and T cells [14]. Progesterone has been reported to have broad anti-inflammatory effects [15]. Finally, androgens, including dihydrotestosterone and testosterone, which are present in higher concentrations in post-pubertal men than women, generally suppress immune cell activity [16], meaning the immune response is dampened in men.

Sex chromosome-linked genes: Female cells contain two X chromosomes, one of which is randomly inactivated during development, whereas male cells contain one X chromosome and one Y chromosome. Leaky expression of inactivated X-chromosome genes can occur, which leads to the upregulation of these genes in women compared with men. Since many genes on the X chromosome regulate the immune function, this phenomenon plays an important role in modulating sex differences in the development of immune-related diseases [17]. These genes code for proteins ranging from Toll-like receptors (TLRs) (for example, TLR7 and TLR8) to cytokine receptors (for example, IL2RG) and transcriptional factors (for example, FOXP3). The Y chromosome also contains numerous regulatory response genes, and Y-chromosome polymorphisms can affect sex-dependent susceptibility to viral infection [18].

## 3. Sex Differences in Cancer Immunotherapy Efficacy

Immune checkpoint inhibitors (including anti-PD-1 antibodies, anti-PD-L1 antibodies, anti-CTLA-4 antibodies, and their combinations) have revolutionized cancer treatment, showing higher efficacy than standard therapies in several cancers, including melanoma [19,20,21,22,23,24,25], non-small cell lung cancer (NSCLC) [26,27,28,29,30,31], head and neck squamous-cell carcinoma [32], and renal cell carcinoma (RCC) [33]. However, the majority of patients do not respond to ICI immunotherapy. As a result, it is of great interest to investigate the factors that influence ICI response. Recently, several meta-analyses were carried out to investigate if sex has a significant effect on immunotherapy efficacy.

ICIs can block the immune inhibitory signals employed by tumor cells and therefore can stimulate the body’s immune response. As explained previously, women generally exhibit a stronger immune environment in their bodies compared to men. This elevated immune response in women may make therapeutic strategies that simply enhance immune response (such as ICIs) less effective in women than in men. This trend has been reported in several meta-analyses reviewed below (Figure 1).

In 2017, Botticelli et al. reported a non-significant trend toward increased benefits for male patients treated with anti-CTLA-4 or anti-PD-1 antibodies. This meta-analysis included 9 randomized controlled trials (RCTs) and 5720 patients (3636 men and 2084 women). Immunotherapy vs chemotherapy was compared in the selected RCTs [34]. In another meta-analysis published in 2018, it was observed that for NSCLC patients, anti-PD1 inhibitors significantly improved the progression-free survival (PFS) in male patients when compared with chemotherapy (hazard ratio (HR) = 0.76; 95% confidence interval (CI) 0.68–0.86); in contrast, women showed no benefit in 5/5 randomized trials (HR = 1.03; 95% CI 0.89–1.20). Five RCTs and 2733 patients (1557 male and 1176 female) were included in this analysis [35]. The interaction between the efficacy of ICIs and patients’ sex was not statistically analyzed in this study.

In 2018, Conforti et al. reported a significant sex difference in ICI vs control trials [36]. Overall, 11,351 patients with advanced or metastatic cancers (7646 men and 3705 women) from 20 RCTs were included in the analysis. The pooled overall survival HR was 0.72 (95% CI, 0.65–0.79) in men and 0.86 (95% CI, 0.79–0.93) in women treated with ICI compared with the control. The difference in efficacy between men and women was significant (*p* = 0.0019); this trend is similar to those reported in previous studies (i.e., men benefit more from ICI treatment than women).

On the other hand, a mouse study reported a different trend. Lin et al. showed that PD-L1 blockade was more effective in treating B16 melanoma in wild-type female mice than male mice. This was due, in part, to the greater ability of anti-PD-L1 antibodies to reduce Treg function in wild-type female mice [37].

In 2019, Wallis et al. reported that there were no significant sex differences in ICI clinical benefits [38]. This meta-analysis included 23 RCTs and 13,721 patients (9322 men and 4399 women). An overall survival benefit of immunotherapy was found for both men (HR = 0.75; 95% CI, 0.69–0.81) and women (HR = 0.77; 95% CI, 0.67–0.88), and the difference between men and women in response to ICI was not statistically significant. This study contained both ICI plus chemotherapy vs control trials and ICI alone vs control trials, whereas the study carried out by Conforti et al. only contained ICI alone vs control trials. As we will discuss later, this difference could explain why Wallis et al. could not identify a significant sex difference in ICI efficacy, whereas Conforti et al. could.

Recently, Conforti et al. continued their previous study and reported the intriguing result that women obtain more clinical benefits from anti-PD1/anti-PD-L1 plus chemotherapy vs control treatment compared to men: females pooled overall survival’s hazard ratio (OS-HR) = 0.44 [95% CI, 0.25–0.76], whereas males pooled OS-HR = 0.76 [95% CI, 0.64–0.91]. In the same study, Conforti et al. also validated their previous finding that men derive more clinical benefits from ICI alone vs control treatment compared with women; females pooled OS-HR = 0.97 (95% CI, 0.79–1.19), whereas males pooled OS-HR = 0.78 (95% CI, 0.60–1.00) [39]. These results may explain why Wallis et al. failed to identify a sex difference in ICI clinical efficacy. This is because the Wallis study contained four RCTs that tested the combination of anti-PD1/PDL1 plus chemotherapy (which were not included in the Conforti study). All four of these trials showed a very large sex-based heterogeneity of efficacy in favor of women; thus, these four RCTs balanced the male-favored positive effects of ICI alone vs control therapy, meaning that, overall, no sex differences were detected in the Wallis study. The reason why ICI plus chemotherapy strategies benefit women more than men may be that chemotherapy can increase the mutational load of tumors and consequently the antigenicity of tumor cells. The already strong immune environment of the female body can then eliminate these tumors with high antigenicity more efficiently than the male body. As we will discuss later in this review, tumors in women generally have lower antigenicity compared to tumors in men.

Recently, there were several other studies supporting a male-favored benefit in ICI therapy. The OS and progression-free survival (PFS) show different trends or significance levels in these analyses for men and women [40]. A recent study has suggested that PFS cannot adequately capture the benefit of PD-1 inhibitors, and OS should be the gold-standard end point for trials involving PD-1 inhibitors [41].

It is important to note that different ICI agents may have different sex-based differences in their efficacy. For example, anti-CTLA-4 antibodies appear to have more significant sex differences than anti-PD-(L)1 antibodies [42,43]. Controversial findings have also been reported in regard to different types of cancer. For example, one study reported that sex differences had a stronger effect on overall survival in melanoma patients vs NSCLC patients [42]. However, another study reported that in melanoma, gender-related factors may not influence the anti-tumor immune response evoked by ICIs [43]. These conflicting findings may be due to sample size or an inherent difference in cancer etiology.

## 4. Sex Differences in Cancer Immunotherapy Predictive Biomarkers

Since the majority of unselected patients will not respond to ICIs, biomarkers for predicting ICI response are urgently needed. Most tumor types show response rates below 40% to PD-1 inhibition; the objective response rates of each tumor type are reported to be highly correlated with the tumor mutational burden (TMB) of each tumor type [44]. In addition to the TMB [45,46], multiple factors are reported to affect ICI effectiveness, including PD-L1 expression [47,48], DNA mismatch repair (MMR) deficiency [49], the degree of cytotoxic T cell infiltration [50], mutational signature [51,52], antigen presentation defects [53,54], interferon signaling [55], tumor aneuploidy [56], T cell gene expression signatures [57], and the microbiota [58]. Currently, efforts are ongoing to identify robust predictive biomarkers to select patients who would derive the maximum potential benefit from immunotherapy.

The TMB, measured as the number of nonsynonymous mutations per megabase sequenced, can be used to predict ICI efficacy and has become a useful biomarker across many cancer types for the identification of patients that will benefit from immunotherapy [30,45,46,59]. Recently, Wang et al. reported that the predictive power of TMB in lung cancer immunotherapy response is influenced by a patient’s sex, with the TMB’s predictive power being significantly better for female than for male lung cancer patients [60] (Figure 1). This study has immediate clinical implications. However, since a limited number of datasets were included in this study, more datasets are needed in the future to further validate this conclusion in both lung cancer and other cancer types. Future development of immunotherapy biomarkers should consider sex differences, and special efforts should be made to improve the performance of immunotherapy-response predictive biomarkers for male lung cancer patients and for patients suffering from other types of cancer.

The female-favored predictive power of the TMB may be due to the fact that female patients have a stronger immune response compared to male patients. Tumors with a high TMB are highly immunogenic, and such tumors exhibit stronger immunosuppressive signals in women than in men (due to the stronger immune environment in women). Immune checkpoint inhibitors block these immunosuppressive signals between tumor cells and immune cells, meaning the strong immune response of women is re-stimulated and the tumor can be attacked more effectively than in males. As a result, ICIs often have a better therapeutic effect in female patients with a high TMB than males.

The concentrations of biomarkers present in patients may depend on the sex of the patient. This is not unexpected for ICI biomarkers such as TMB and microbiota [61,62,63]. Ramsey et al. analyzed the levels of over 170 protein and small-molecule biomarkers in men and women with varying hormonal statuses and reported that the concentration of 56% of biomarkers varied between men and women [64]. However, the sex difference in the predictive power of biomarkers is highly intriguing, as essentially no other studies report sex differences in the predictive power of cancer immunotherapy biomarkers; thus, this revelation points to a novel field of study. As a result, the predictive power of biomarkers, specifically immunotherapy-related biomarkers, should be analyzed considering the sex of the subjects.

## 5. Sex Differences in Tumor Antigenicity

TMB reflects the antigenicity of tumors, as extra mutations in the genome will encode neoantigens. In melanoma, Gupta et al. reported a statistically significantly greater burden of missense mutations among men (male-to-female ratio = 1.85, 95% CI = 1.44–2.39) [65]. Similarly, in lung adenocarcinoma, male tumors harbored a statistically greater burden of genetic alterations than female tumors (male-to-female ratio = 1.636, 95% CI = 1.343–1.992) after adjusting for age at the time of surgery, stage, and smoking status [66]. In a TCGA (The Cancer Genome Atlas) pan-cancer analysis, male-derived tumors exhibited a significantly higher density of somatic-coding single nucleotide variants (SNVs) than female-derived tumors in both univariate analysis and multivariate analysis, adjusting for imbalances in sample numbers across tumor type, race, and age [67]. These studies suggest male tumors have a higher TMB than female tumors.

In addition to the TMB, other cancer-associated antigens also show sex differences. Cancer germline (also known as cancer testis) antigen genes are normally expressed in germ cells and trophoblast tissues and are aberrantly expressed in a variety of human malignancies. Cancer germline antigens are potential targets for cancer vaccines due to their restricted expression in neoplastic cells. Shigematsu et al. have shown that the cancer germline antigen MAGE-A4 is expressed more frequently in male than in female NSCLC patients [68]. Additionally, Gure et al. reported that the expression of the cancer germline antigens NY-ESO-1, LAGE-1, MAGE-A1, MAGE-A3, MAGE-A4, MAGEA10, CT7/MAGE-C1, SSX, and SSX4 is strongly correlated with the male sex in NSCLC patients [69]. Sex differences in the expression of cancer germline antigens in other cancer types are still waiting to be studied.

Altogether, current research suggests that male tumors tend to have a higher TMB and more cancer germline antigens than female tumors. This reveals that, generally, male tumors are more antigenic than female tumors (Figure 1). This is consistent with the fact that the female body mounts a stronger immune response than the male body, meaning tumors of high antigenicity are less likely to persist in female patients. The reduced antigenicity of female tumors could lead to the compromised immunotherapy efficacy observed in female patients.

## 6. Sex Differences in Immune-Related Adverse Events

Through enhancing the activity of the immune system, ICIs can have inflammatory side effects, called immune-related adverse events (irAEs), which mainly involve the gastrointestinal tract, skin, endocrine glands, liver, and lungs but can potentially affect any tissue [70,71]. Different ICI agents may have distinct irAEs. For example, CTLA-4 inhibits the immune response in several ways, including attenuating T cell activation at a proximal step in the immune response [72], whereas PD-1/PD-L1 is generally believed to suppress T cells at later stages of the immune response in peripheral tissues [73,74]. The distinct functions of CTLA-4 and PD-1 are reflected in the different phenotypes seen in knockout mouse models: The mice lacking the CTLA-4 gene die from lymphoproliferation and fatal multiorgan tissue destruction [75,76], whereas the mice lacking PD-1 are variable, showing autoimmune diseases [77].

Generally, women have more adverse events from chemotherapy than men [78,79], but women also have increased response rates and survival after chemotherapy compared to men [80,81]. Recently, Joseph et al. reported that women experienced an increased risk of severe symptomatic adverse effects and objective adverse effects after immunotherapy [82]. Additionally, Duma et al. reported that women may be at a higher risk of irAEs compared with men when treated with anti-programmed cell death protein 1 therapy. They also reported that women are more likely to develop certain irAEs, including endocrinopathies and pneumonitis [83].

Altogether, recent studies suggest that there are sex differences in irAEs, with women having a higher chance of exhibiting irAEs (Figure 1). The detailed irAEs for specific ICI agents and cancer types should be investigated in depth, and this information will be helpful for clinicians to diagnose these treatment-related complications early, potentially reducing their associated morbidity and mortality.

## 7. Conclusions and Perspectives

After years of study, it is now clear that women generally mount a stronger immune response compared to men. Sex hormones (including estrogen, progesterone, and androgens) and sex-chromosome-related genes are the main factors driving these sex differences in immunity. Cancer immunotherapy, represented by ICIs, enhances the body’s immune response to kill tumor cells. Sex-related differences in immune response should not be neglected in immunotherapy design and analysis. In cancer immunotherapy clinical trials, women should be sufficiently represented, as the clinical effects observed in men do not necessarily also occur in women, and vice versa. The clinical design of cancer-immunotherapy-response prediction biomarkers should also consider sex differences; the biomarker TMB is preferentially useful in female patients, while the predictive power of PD-L1 does not show a sex difference. Furthermore, immunotherapy-related adverse events also show sex differences, and this can potentially influence the clinical management of immunotherapy-treated patients.

Recent studies suggest that male patients obtain more benefits from ICIs vs control compared with female patients; however, female patients tend to obtain more clinical benefits from ICI plus chemotherapy vs control treatment compared to male patients. This indicates that the design of immunotherapy strategies should be optimized on the basis of patients’ sex. The inherent strong immune response in the female body means that therapies that simply enhance the immune response are less effective in females than in males; this explains why ICIs on their own are less effective in females. However, ICI plus chemotherapy, or therapy that enhances the antigenicity of tumor cells, is more effective in females compared to males.

Based on the above evidence, here we propose that immune microenvironment-enhancing strategies may be more suitable for male patients, whereas tumor-cell antigenicity-enhancing strategies may be more suitable for female patients (Figure 1). How can the immune microenvironment be enhanced? Hormone therapy and cytokine therapy can boost the body immune response; for example, androgen deprivation therapy in prostate cancer has been reported to be able to stimulate the immune response [84], and this has been combined with ICI in clinical trials [85]. How can tumor antigenicity be enhanced? Radiation therapy and DNA-damaging chemotherapy can cause mutations in cancer and consequently increase the antigenicity of tumor cells. Cancer germline antigens could also be stimulated to enhance tumor cell antigenicity.
